# Temporal Trend in Hospitalizations for Acute Diabetic Complications: A Nationwide Study, Italy, 2001–2010

**DOI:** 10.1371/journal.pone.0063675

**Published:** 2013-05-23

**Authors:** Flavia Lombardo, Marina Maggini, Gabriella Gruden, Graziella Bruno

**Affiliations:** 1 National Institute of Health, Rome, Italy; 2 Department of Medical Sciences, University of Turin, Turin, Italy; Sapienza University of Rome, Italy

## Abstract

**Background:**

We investigated temporal trends and geographic variations in both hospitalizations and in-hospital mortality rates for acute diabetic complications (ADC) in the Italian universal health care system.

**Methods and Findings:**

A retrospective review of the medical records of patients with either primary or secondary discharge diagnosis of hyperglycaemic acute complications (ICD-9-CM codes 250.1, 250.2, 250.3) or hypoglycemic coma (ICD-9-CM code 251.0) was performed in period 2001–2010. Standardized rates by age and gender on 2001 Italian population and by diabetic population were calculated. We identified 7,601.883 diabetes-related hospital discharges. Out of them, 266,374 (3.5%) were due to ADC, either ketoacidosis/hyperosmolarity (94.4%) or hypoglycemic coma (5.6%). The rate of discharge for ADC decreased by 51.1% from 2001 to 2010 (14.4 vs. 7.1 discharge rate/1,000 diabetic people; 5.7% decrease per year, test for trend, p<0.001) with a similar trend for both hyperglycemic and hypoglycemic complications. Diabetic people in the younger age groups (≤19 and 20–44 years old) had a significantly greater rate of discharge for ADC than people aged 65 years and over (≤19 10-fold increase; 20–44: 2-fold increase). In-hospital mortality rate was 7.6%, with 211 preventable deaths in younger diabetic people (≤44 years old). There was a large variability among Italian Regions and the ratio between the highest and the lowest regional discharge rate reached 300% in 2010.

**Conclusions:**

Decreasing temporal trend in hospitalizations for preventable ADC suggests improving outpatient care. In younger diabetic patients, however, both hospitalization rates and in-hospital mortality are still a matter of concern.

## Introduction

Intervention studies have convincingly demonstrated the importance of maintaining a good glycemic control over time to decrease the incidence of micro- and macrovascular complications in both type 1 and type 2 diabetic patients [1]–[3]. Severe hypoglycemia, however, is a major side effect of glucose-lowering therapy and there is concern about the potential risk of both cardiovascular events and mortality in patients experiencing severe hypoglycemic episodes [4]–[7]. Self-monitoring of blood glucose (SMBG) has been increasingly used by diabetic patients over the last decade; however, subgroups of diabetic patients, such as children, adolescents, and elderly with comorbidities, are still more likely to experience both hypo- and hyperglycemic acute diabetic complications [8]–[9]. Indeed, ketoacidosis and hyperosmolarity are the leading cause of mortality in both children and young adults with type 1 diabetes [10]–[11], and both low socioeconomic level and poor education increase the risk of these events [12]. Moreover, in type 2 diabetes, acute hyperglycemic complications may represent precipitating factors as well as major consequences of associated comorbidities, such as cognitive impairment and cardiovascular diseases.

Hospital admission for acute diabetic complications is often avoidable and mortality associated with these events are sentinel health events of inadequacy of outpatient care [13],[14]. Improving diabetes clinic accessibility to patients of different age groups and socioeconomic levels as well as increasing their involvement in educational programs may reduce the risk of acute diabetic complications. Despite the relevance of this issue, few epidemiological studies have assessed changes in hospital admission rates for acute diabetic complications over time and space. In addition, most of the available data are limited by the recruitment of either people cared for in a single centre or children and adolescents only [15]–[23].

In Italy the National Health System (NHS) provides universal health coverage for all diabetic patients. In this nationwide study, we studied the temporal trend in hospitalizations rates for both acute hyperglycemic complications (ketoacidosis/hyperosmolarity) and hypoglycemic coma in the period 2001–2010 and trend differences among Italian regions. We also assessed if trends in both hospitalizations and in-hospital mortality rates were heterogeneous among age groups.

## Methods

Hospital admission data (2001–2010 period) were obtained from the Italian National Hospital Discharge Database as anonymous individual records, reporting both administrative and clinical data. The National Hospital Discharge Database, which was established in 1994, collects complete data on hospitalizations from both public and private Italian hospitals. Data include age, gender, region of residence, date of admission, length of hospital stay, primary and up to five secondary diagnoses (coded based on the ICD-9-CM), and discharge dispositions. Neither ethical approval nor individual written consent by adults and children patients were requested according to Italian law (anonymous aggregated data). Hospital admission for diabetic ketoacidosis/hyperosmolar coma were defined as those reporting the 250.1, 250.2, or 250.3 ICD-9-CM codes as primary or secondary discharge diagnosis. Diagnosis of both hypoglycemic coma (ICD-9-CM code: 251.0) and diabetes (ICD-9-CM code: 250) were required to define the patient as admitted for hypoglycemic coma. Hospitalizations for either pregnancy or puerperium (Diagnosis Related Group DRG: 370–384) and childbirth (DRG: 385–391) were excluded from the study. Re-hospitalizations were determined using a unique and anonymous identification code. The accuracy of ICD-9-CM coding improved over the study period and the proportion of hospital discharges without a unique identifier declined from 7.5% in 2001 to 1.7% in 2010. Hospitalization rates were calculated every year as the ratio between the numbers of hospital discharges and the resident populations per 100,000, standardized by both age and gender on the Italian population in 2001. Population figures by gender, age, calendar year, and Region were obtained from the National Statistical Office (ISTAT). Age- and sex-specific hospitalization rates for diabetic patients were calculated using the Italian age- and sex-specific diabetes prevalence published by ISTAT and based on annual national surveys involving a sample of about 50,000 families, representative of the Italian population. Estimates of diabetes prevalence were accurate in the older age groups, with a relative error around 2%, while in the youngest age groups (0–19 years) were affected by large annual variations, due to the lower numbers of persons on which estimates were based. Ninety-five per cent confidence intervals of specific rates were calculated considering the sampling error of diabetic population estimates. Temporal trends in hospital admission rates were assessed through the chi-squared for trend. In-hospital deaths were identified selecting all admissions for acute diabetic complications with death as discharge disposition. In-hospital mortality rates were calculated dividing deaths by the number of patients hospitalized for acute complications, after exclusion of discharges without patient identification code. Mortality rates were calculated both yearly and on the whole ten-year period. Data analysis was performed using STATA, version 11 (Stata Corporation LP, College Station, TX, USA).

## Results

Out of the 7,601.883 diabetes-related hospital admissions in the period 2001–2010, 266,374 (3.5%) were due to acute diabetic complications and occurred in 214,899 patients (24,153 mean annual number) (Table 1). Diabetic ketoacidosis/hyperosmolar coma was the predominant reason for hospital admission (94.4%) and solely 5.6% of admission were for hypoglycemic coma. There were no differences between genders in admission rates. Mean age at hospital admission was 65.8±20.4 years (62.7±29.2 in men and 68.6±20.1 in women, p<0.001) without significant changes over the study period. However, age at hospital admission for hypoglycemic coma increased from 71.5±16.0 in 2001 to 74.6±15.0 years in 2010 (p<0.001). Hypoglycemic coma was the primary diagnosis in 80.2% of discharges. Diabetic ketoacidosis/hyperosmolar coma was the primary diagnosis in only 39.0% of discharges, but there were significant differences among age groups (0–19: 91.1%, 20–44: 68.1%, 45–64: 34.3%, 65 years and over: 28.8%).

**Table 1:** Hospitalizations for acute diabetic complications in Italy, 2001–2010.

	Acute diabetic complications	Acute hyperglycemic complications	Hypoglycemic coma
**Discharges**			
N	266,374	251,528	14,846
Acute diabetic complications as primary diagnosis, n (%)	110,033 (41.3%)	98,133 (39.0%)	11,900 (80.2%)
Duration of stay in days, mean (SD)	9.2 (11.1)	9.3 (11.3)	6.9 (6.8)
Age distribution (years)			
0–19	8.3%	8.7%	1.1%
20–44	9.0%	9.2%	6.0%
45–64	20.6%	21.0%	14.0%
65+	62.1%	61.1%	78.9%
**Patients**			
N	214,899	203,273	13,764
Female, n (%)	111,917 (52.1%)	105,321 (51.8%)	7724 (56.1%)
Age*, mean (SD)	65.8 (20.4)	65.3 (20.6)	72.8 (15.4)
Rehospitalizations in the study period, n (%)	26,552 (12.4%)	24,629 (12.1%)	543 (3.9%)
In-hospital deaths, n (%)	16,402 (7.6%)	16,117 (7.9%)	285 (2.1%)

* Age at first discharge in the study period.

Standardized hospitalization rates for acute complications decreased 42.5%, (56.3/100,000 in 2001 vs. 32.4/100,000 in 2010) over the all study period (Table 2) with a 4.7% decline per year (test for trend, p<0.001) that was similar for both hyperglycemic and hypoglycemic complications. The decreasing trend with respect to the diabetic population was 51.1% (5.7% per year, test for trend, p<0.001). In the same period, hospitalization rates for all causes (discharges with diabetes as either primary or secondary diagnosis) decreased by 28.9% only (333.0/1,000 diabetic people in 2001 vs. 236.7/1000 diabetic people in 2010; test for trend, p<0.001).

**Table 2:** Hospital admission rates for acute diabetic complications in Italy, 2001–2010.

	Acute diabetic complications			Acute hyperglycemic complications			Hypoglycemic coma		
	N	rate/100,000 residents*	rate/1,000 diabetic people (95% CI)	n	rate/100,000 residents*	rate/1,000 diabetic people (95% CI)	n	rate/100,000 residents*	rate/1,000 diabetic people (95% CI)
**2001**	32,096	56.3	14.4 (13.8–15.1)	30,302	53.2	13.6 (13.1–14.3)	1,794	3.1	0.81 (0.84–0.77)
**2002**	30,304	53.1	13.7 (13.1–14.3)	28,546	50.0	12.9 (12.4–13.5)	1,758	3.1	0.80 (0.76–0.83)
**2003**	30,072	51.7	13.5 (12.9–14.1)	28,457	49.0	12.7 (12.2–13.3)	1,615	2.8	0.72 (0.69–0.76)
**2004**	27,694	46.9	11.9 (11.3–12.4)	26,202	44.4	11.2 (10.7–11.7)	1,492	2.5	0.64 (0.61–0.67)
**2005**	26,861	44.7	11.0 (10.5–11.6)	25,395	42.3	10.4 (9.9–10.9)	1,466	2.4	0.60 (0.57–0.63)
**2006**	26,512	43.5	10.2 (9.7–10.7)	25,067	41.2	9.6 (9.2–10.1)	1,445	2.3	0.56 (0.53–0.58)
**2007**	25,177	40.7	9.3 (8.9–9.7)	23,714	38.4	8.7 (8.4–9.1)	1,463	2.3	0.54 (0.52–0.56)
**2008**	24,732	39.3	8.6 (8.3–9.0)	23,361	37.2	8.2 (7.8–8.5)	1,371	2.1	0.48 (0.46–0.50)
**2009**	22,052	34.5	7.7 (7.3–8.0)	20,777	32.6	7.2 (6.9–7.5)	1,275	1.9	0.44 (0.42–0.46)
**2010**	20,874	32.4	7.1 (6.8–7.4)	19,707	30.6	6.7 (6.4–7.0)	1,167	1.7	0.39 (0.38–0.41)
***Δ%*** **		*−42.5*	*−51.1*		*−42.4*	*−51.1*		*−45.1*	*−51.7*

* Standardized by age and gender on 2001 Italian population.

** Relative percentage variation from 2001 to 2010.

**Figure 1:**
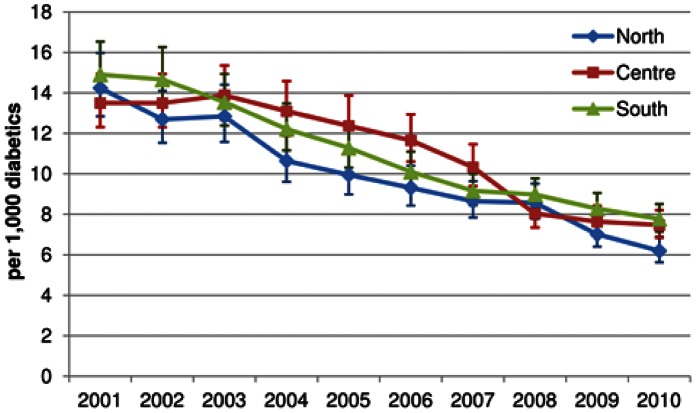
Temporal trend in hospitalization rates/1,000 diabetic people for acute diabetic complications, by North, Center and South of Italy, 2001–2010. Vertical bars indicate 95% CIs.

Table 3 shows hospital admission rates/1,000 diabetic patients by age group and calendar year. Compared to diabetic patients aged 65 years and over, those aged 0–44 years showed a 4-fold higher risk of hospitalization (10-fold in the age group 0–19 years and two-fold in the age group 20–44 years). The decreasing trend over calendar period, however, was quite similar among age groups and genders. Similar decreasing trends over time were observed in Northern, Central, and Southern Italy. Admission rates per 1,000 diabetic persons in 2010 were 6.2 (95% CI, 5.6–6.9) in Northern, 7.5 (95% CI, 6.9–8.2) in Central, and 7.8 (95% CI, 7.2–8.5) in Southern Italy (Figure 1). Large variability of rates was found, however, among Italian Regions, with the lowest rate per 1000 diabetic persons of 3.8 (95% CI, 3.3–4.4) and the highest rate of 10.8 (95% CI, 9.2–13.0).

**Table 3:** Hospital admission rates per 1,000 diabetic people for acute diabetic complications, by age groups, 2001–10, Italy.

	Rates/1,000 diabetic people (95%CI)		
	Age 0–44 years	Age 45–64 years	Age 65+ years
**2001**	48.3 (37.9–66.7)	10.2 (9.3–11.3)	13.9 (13.1–14.8)
**2002**	43.9 (34.7–59.7)	9.5 (8.7–10.4)	13.5 (12.8–14.4)
**2003**	40.8 (32.6–54.7)	9.9 (9–10.9)	12.9 (12.2–13.7)
**2004**	36.8 (29.4–49.2)	8.9 (8.2–9.9)	11.1 (10.5–11.8)
**2005**	33.7 (27.0–45.0)	7.8 (7.1–8.6)	10.5 (9.9–11.2)
**2006**	34.4 (27.5–45.7)	6.6 (6.1–7.3)	9.9 (9.4–10.5)
**2007**	36.9 (29.4–49.5)	5.9 (5.4–6.5)	8.8 (8.3–9.3)
**2008**	26.2 (21.5–33.5)	6.5 (6.0–7.2)	8.0 (7.6–8.5)
**2009**	27.8 (22.6–36.3)	5.3 (4.8–5.8)	7.2 (6.8–7.6)
**2010**	23.8 (19.9–29.6)	5.0 (4.6–5.5)	6.5 (6.2–6.9)
***Δ%*** *	*−50.8%*	*−51.1%*	*−53.1%*

* Relative percentage variation from 2001 to 2010.

Rehospitalizations for acute diabetic complications over the 2001–2010 period occurred in 12.4% of patients (12.1% for hyperglycemic complications and 3.9% for hypoglycemic coma). Age at first hospital admission of these patients was 57.1±24.9 for hyperglycemic complications and 66.9±17.2 years for hypoglycemic coma.

In the study period, 16,812 in-hospital deaths were recorded among diabetic patients admitted for acute complications and 16,402 were included in the mortality analysis, giving an in-hospital mortality rate of 7.6% over the study period, without significant variations over time. In-hospital mortality rate for hypoglycemic coma only showed a remarkable, but not statistically significant, increase in 2010 compared to 2009 (3.1% vs 1.9, p = 0.06). Out of 211 deaths that occurred among patients aged 0–44 years, only one was due to hypoglycemic coma and occurred in an adult diabetic patient.

## Discussion

This is the one of the few studies examining hospital admissions rates for acute diabetic complications in diabetic people and the first nationwide study covering a long time period. We provide evidence that hospitalizations for acute diabetic complications decreased by 51% in diabetic patients over a ten years period, and that the extent of the reduction was similar for hypoglycemic coma and acute hyperglycemic complications, whereas the reduction in hospitalizations rates for all causes was more limited (−28.9%). This finding is of relevance as it suggests an overall improvement over time in quality of diabetes outpatient care in Italy. However, in-hospital mortality did not improve over time as yet in 2010, 7% of patients with acute hyperglycemic complications, and 3% of those with hypoglycemic coma died during their hospitalization. Second, during the study period the highest hospitalization rate was found in the youngest diabetic patients. In spite of similar decrease over time among age groups, diabetic people aged 19 years and less and those aged 20–44 years maintained a ten-fold and two-fold higher admission rates for preventable acute complications, respectively, than those aged 65 years and over, mainly due to ketoacidosis. Among diabetic patients aged 44 years and lower, in-hospital mortality accounted for as high as 211 preventable deaths in the period 2001–10. Finally, although the decreasing trend was similar in Northern, Central, and Southern Italy, admission rates remained higher in 2010 in the latter. A three-fold difference among Italian Regions was evident, which cannot be accounted for by differences in prevalence of the disease and might indicate inequalities of care under the universal coverage of NHS.

Our results are consistent with studies showing similar declining risk of hospitalizations for acute diabetic complications in the period 1994–99 in Canada [15], and 1998–2006 in the United States, though in the latter the decrease was limited to diabetic patients aged 45 years and over, while rates remained steadily high in the youngest patients [16]. In our study, the decline was 5.7% per year, with more than one half reduction over the ten years period and similar among age groups and genders. Several factors may be involved in the decreasing temporal trend of hospitalizations for acute complications in different countries, including higher feasibility of antihyperglycemic medications in more recent years and widespread employment of SMBG. Both an improvement in metabolic control over time and an increased use of both new drugs and insulin analogues have been observed in Italy, which might be related, at least in part, to our results [24],[25]. However, we have also recently shown that as high as 30% of a large representative population-based cohort had no HbA1c measurement over the previous 12 months [26] and this disappointing finding may be related to the persisting high hospital admissions rate for acute complications in 2010, in spite of a decreasing trend over time.

A particularly disappointing finding was found in diabetic people aged 19 years and lower, who yet in 2010 experienced a ten-fold higher admission rate for acute complications than diabetic patients aged 65 years and over, almost entirely due to ketoacidosis. Consistently, a study conducted in Scotland showed that people with type 1 diabetes experienced a large number of hospital admissions for diabetic coma [22]. Socioeconomic factors may be involved in the admission rates for acute diabetic complications [8]–[14],[15],[22]. In a German study a three-fold higher risk of severe ketoacidosis requiring hospitalization at onset of type 1 diabetes was found in children with lower socioeconomic level [12]. In Finland, a persistently high mortality rate for acute complications was evident in adult-onset type 1 diabetes only, whereas in childhood-onset diabetes there was a parallel decline, suggesting that psychological support to adolescent and young adults might allow to increase their compliance to treatment and reduce mortality risk [10]. In the United States, no variations of in-hospital mortality rates for preventable acute complications were found in young diabetic patients only [16]. In our survey, as high as 211 preventable in-hospital deaths in people aged 44 years and lower were identified over the ten-year period, almost entirely due to ketoacidosis, with no variation of mortality rates over time.

Most of admissions for acute complications have been registered in people aged 65 years and over, who represents two third of prevalent diabetic people in Italy [24], and negatively impact on costs of the disease. With respect to non-diabetic people of similar age and sex, direct costs of diabetic people are 4-fold higher, and hospitalizations account for 50% of this amount [26]. Although acute complications represents 3.5% only of all diabetes-related hospitalizations, their reduction will contribute to decrease the cost burden of health care delivery in diabetes. Elderly diabetic patients are generally characterized by comorbidities that may act as precipitating factors as well as major consequences of acute diabetic complications. This may explain the higher proportion of discharged elderly with hyperosmolarity defined as secondary rather than primary diagnosis. Prevention through appropriate education, improved self-care and compliance remains the most important aspect of managing acute diabetic complications.

Our study relies on national hospital discharge data, which can be currently considered complete. As we have identified all hospital admissions with diabetes listed as one of the discharge diagnoses, our data may have underestimated hospitalization rates due to the proportion of admitted diabetic patients who were discharged without mentioning diabetes in discharge records. In Italy remuneration of hospital activities is regulated by the DRG’s system, and guidelines have been implemented to increased physicians expertise on appropriate filling of discharge records. As regards diabetes, it is generally emphasized to include the disease as secondary rather than primary diagnoses, due to its lower remuneration with respect to other diseases. Our selection procedures including acute complications defined as either primary or secondary discharge causes should have allowed to capture most if not all discharges.

In conclusion, this nationwide study showed that hospitalizations for acute diabetic complications decreased by 51% in diabetic patients over a ten-year period, similarly among age groups and for hypoglycemic coma and acute hyperglycemic complications. In-hospital mortality, however, did not improve over time. Moreover, the youngest diabetic patients still experienced the highest hospitalization rates for acute complications, mainly ketoacidosis, with relevant numbers of preventable deaths over ten-years period. Geographical variations within Italy might be related to selective lower accessibility to care in Italian areas.
